# Recent advances in the understanding and psychological treatment of social anxiety disorder

**DOI:** 10.12703/r/12-8

**Published:** 2023-04-14

**Authors:** Kate Wolitzky-Taylor, Richard LeBeau

**Affiliations:** 1 Department of Psychiatry, University of California, Los Angeles, CA, USA; 2 Department of Psychology, University of California, Los Angeles, CA, USA

**Keywords:** Social anxiety, cognitive behavioural therapy

## Abstract

Social anxiety disorder (SAD) is characterized by persistent anxiety or avoidance of social situations because of a fear of negative evaluation. Cognitive behavioral therapy (CBT) (typically with an exposure component) is a first-line treatment for social anxiety, but there remains room for improvement with regard to treatment efficacy. Therefore, the field continues to better understand the mechanisms underlying SAD and its common and complex comorbidities in order to develop targeted interventions to improve symptom outcomes. Additionally, efforts are under way to improve the efficacy and accessibility of CBT. This review outlines major advances in understanding and treating SAD in adults over the past roughly 3 years (2019 to early May 2022). Themes are identified and discussed, as are recommendations for future research.

## Current status of social anxiety disorder and its treatment

The diagnostic classification and criteria for social anxiety disorder (SAD), in contrast to those of many other prevalent disorders, have gone largely unchanged in recent decades. However, that is not to imply that research on SAD has been stagnant. In reality, an astonishing amount of cutting-edge research on SAD has been conducted in recent years. Such research is necessitated by the fact that SAD remains a prevalent condition, whose estimated 12-month prevalence in the United States is 8%^1^. It is also a markedly disabling condition with an early onset^2^ and persistent course^3^. SAD is associated with significant impairment in social, occupational, and academic domains as well as decreased quality of life^4^.

Fortunately, several effective treatments for SAD exist. The gold-standard psychosocial treatment for SAD is cognitive behavioral therapy (CBT) delivered in individual or group format^5–7^. Increasing evidence also supports the effectiveness of acceptance and commitment therapy (ACT) for SAD^8–10^. Various medications, particularly selective serotonin reuptake inhibitors (SSRIs), have been shown to be effective for SAD as well^5^. However, the ability to access evidence-based treatments remains a challenge^11,12^. Even among those who are able to access evidence-based treatments, many fail to respond to treatment or relapse after completing treatment^13^. Thus, ongoing research is needed to figure out how to maximize the accessibility and effectiveness of current treatments as well as to develop novel treatments.

The purposes of this review were to provide a thematic glimpse into the most recent developments in the understanding and management of SAD and to guide researchers and clinicians to particularly important articles for a more in-depth subsequent review. We focused on this broad topic from a psychological lens. Although we acknowledge the many contributions from the fields of neuroscience, psychiatry, and pharmacology/pharmacogenetics, as well as child and adolescent psychology and psychiatry, we discuss advances in our understanding of the psychological processes underlying SAD and of behavioral (and cognitive) treatment advances for SAD in adults. Importantly, we also discuss the recent emphasis on understanding SAD and its treatment in the context of common and complex psychological comorbidities.

## Methods for this review

Our review consisted of PubMed and Google Scholar searches for articles from 2019 to 2022 with the following search terms: “social anxiety disorder,” “social anxiety disorder treatment,” “social anxiety etiology,” and “social anxiety assessment.” We conducted an extensive review of abstracts that appeared and then we selected articles that reported on the relevant themes of this review. Review articles and meta-analyses that were published between 2019 and 2022 but that synthesized findings from previously published research were excluded. Note that the review was conducted in spring 2022, so any studies published after the first week of May 2022 were not reviewed.

## Recent advances in improving the understanding of SAD

### Factors contributing to the onset and maintenance of SAD

Recently, a great deal of research has been conducted on factors that are related to the onset and maintenance of SAD. A brief overview of the research conducted on a large array of constructs is provided here. A notable area of recent research involves examining the role of adverse social experiences in the development of SAD. Bjornsson and colleagues^14^ examined “social trauma,” events which involve humiliation and rejection and would not necessarily be categorized as Criterion A traumatic stressors, because they were not life-threatening. They found that social trauma was highly prevalent among individuals with SAD and that nearly one third of their sample experienced clinically significant post-traumatic stress disorder (PTSD) symptoms related to their social trauma. This work highlights the potential clinical importance of understanding socially traumatic events and the possible limitations of our traditional definitions of what constitutes a trauma. Brühl and colleagues^15^ examined adverse childhood experiences and found that adverse experiences that were social in nature were not uniquely associated with SAD relative to other mood or anxiety disorders. Although contrary to expectations, this study was limited by being both retrospective and cross-sectional. This limitation is frequently observed in research that tries to elucidate the relationship between adverse early experiences and later psychopathology. Longitudinal research studies that are designed to examine not only *which* adverse experiences (e.g., social trauma) are associated with SAD onset but *how they relate to SAD onset* and whether they are uniquely associated with SAD onset (or more broadly associated with onset of psychopathology) are needed.

A great deal of research has been conducted on cognitions, emotional states, and behaviors that may serve as maintenance factors for SAD. Most of these studies were conducted with relatively small, non-clinical samples in laboratories. Oren-Yagoda, Schwartz, and Aderka^16^ analyzed data from a daily experience study and found that individuals with social anxiety experience higher levels of envy than those without social anxiety and that experiences of envy (particularly in social contexts) uniquely predicted subsequent anxiety. Arch and colleagues^17^ found that individuals with SAD had more frequent off-task thoughts and rated these thoughts as less controllable, more personal, and associated with worse mood than those without social anxiety. Bailey and colleagues^18^ found that perseverative cognition was highly prevalent among individuals with SAD and was associated with lower heart rate variability following a negative social interaction. Goodman and colleagues^19^ found that although individuals with SAD derived less pleasure from social interactions than those without, they still derived positive emotions from social interactions and experienced more positive emotions than they did from non-social situations. Maleki and colleagues^20^ found that affective theory of mind (i.e., ability to infer others’ emotional states and feelings) but not cognitive theory of mind (i.e., ability to infer others’ beliefs and intentions) was disrupted in individuals with SAD relative to healthy controls. Romano, Tran, and Moscovitch^21^ found evidence of context-specific memory impairments among individuals with SAD for social situations with positive outcomes.

Additionally, new research has been conducted on the role of behavioral avoidance^22^, safety behaviors^23^, attentional bias to threatening faces^24^, gaze avoidance^25^, delays in attention disengagement from social threat^26^, rumination^27,28^, blushing^29^, inattention, intolerance of uncertainty^30^, and fearful attachment style^31^.

Although the studies described above utilized different methods and focus on different cognitions, emotional states, and behaviors, they all provide interesting examples of how theoretically driven research can help elucidate key maintenance factors in SAD. The identification of such factors is essential for researchers to develop novel treatments and refine and enhance our existing treatments to ensure that they are effectively and efficiently targeting key mechanisms through which symptom reduction can occur. These studies also underscore the need for treatment studies to include measures (both self-report and more objective measures) of hypothesized mechanisms in their assessment batteries. Greater inclusion of such measures in treatment trials would also help overcome some of the limitations caused by relying on small, clinical analogue samples for mechanism research. It is important that future research seek to elucidate which of these constructs are unique to SAD versus, more broadly, characteristics of anxiety disorders or all mental disorders, as such knowledge is useful for treatment development (particularly the development of treatments for comorbid conditions and transdiagnostic treatments).

### Factors contributing to impairment in SAD

Several research investigations in recent years have continued to examine the high levels of impairment experienced by many individuals with SAD. A well-designed investigation by Vilaplana-Pérez and colleagues^32^ utilized data from over 2 million individuals born in Sweden and presented some of the most compelling and nuanced data to date regarding markedly worse educational performance across the life span for individuals with SAD relative to those without. The effect was particularly prominent for females. Impressively, their findings remained significant after controlling for psychiatric comorbidity and a host of genetic and social factors (utilizing data from siblings without SAD). Peyre and colleagues^33^ utilized data from a large, nationally representative epidemiological survey to examine the relationship between age of onset of SAD and functioning. Interestingly, they found that a later age of onset was associated with greater psychiatric comorbidity and diminished quality of life. Krygsman and Vaillancourt^34^ analyzed data from over 700 adolescents and found that those who had a trajectory of high and increasing social anxiety symptoms from 5th to 12th grade were much more likely than those with moderate and low symptom trajectories to meet criteria for major depression and cannabis use disorder as adults. Investigations such as these leverage large samples and sophisticated analytical methods to improve our understanding of the factors that contribute to worse outcomes for individuals with SAD and help us figure out what subgroups might need additional intervention and the type of additional interventions they may need (e.g., educational interventions for female children and adolescents with SAD). Future research should also seek to disentangle the degree to which observed impairments result from SAD as opposed to conditions comorbid with SAD to improve our understanding of where prevention and intervention efforts are most needed.

### Understanding SAD comorbidity

Numerous studies that seek to elucidate our understanding of SAD comorbidity have been conducted in recent years. Two notable studies examined the comorbidity between SAD and alcohol use disorder (AUD) and came to markedly different conclusions. Torvik and colleagues^35^ analyzed a longitudinal dataset of 2,801 Norwegian pairs of twins and found that SAD had a direct effect on the development of AUD that was not found for other anxiety disorders. In contrast, Miloyan and van Doorn^36^ analyzed data from two nationally representative longitudinal datasets and found that neither lifetime subclinical social fears nor full SAD was associated with later AUD.

Wong^37^ applied sophisticated statistical methodology to data from outpatients with early psychosis and found a direct relationship between the severity of negative symptoms and ideas of reference with social anxiety symptoms that provides greater understanding for the frequently observed comorbidity between social anxiety and psychosis. Langer and colleagues^38^ conducted network modeling to better understand the well-established comorbidity between SAD and major depressive disorder (MDD) and concluded that the comorbidity likely results from direct relationships between associated nodes as opposed to hallmark symptoms of the disorder. Frandsen and colleagues^39^ examined the relationship between SAD and avoidant personality disorder (APD) and concluded that although SAD is associated with lower levels of interpersonal distress than APD, SAD is related with higher levels of phobic anxiety than APD. They also concluded that both disorders feature non-assertiveness as a core feature.

Recent research has also sought to understand the relationship between SAD and suicidality. Chung and colleagues^40^ analyzed data from a large sample of individuals with SAD and found support for the interpersonal theory of suicide^41^, according to which perceived burdensomeness and thwarted belongingness were significantly predictive of acute suicidal ideation in individuals with SAD over and above the effects of depression. Duffy and colleagues^42^ analyzed data from individuals with social anxiety and found that perceived burdensomeness but not thwarted belongingness was associated with previous two-week suicidal ideation over and above the effects of depression. It is plausible that the divergence in findings was related to markedly different time frames for the suicidality assessment between the two studies. Yarrington and colleagues^43^ examined suicidality in a large and diverse sample of individuals with SAD who were seeking community employment services. The authors found that suicidal ideation and a history of suicide attempts were highly prevalent but that they were largely accounted for by depression comorbidity, which was experienced by virtually all of the sample.

Taken together, these studies have contributed to a deeper understanding of SAD comorbidity and, in several cases, elucidate the explanatory variables that account for the comorbidity. However, given some of the discrepant findings described above, additional research is needed to ascertain whether an accumulation of more data will point to more consistent findings in one direction or another (e.g., for the role of social anxiety in contributing to AUD onset). Research on SAD comorbidity is also critical to helping us better understand what clinical features are unique to SAD and which are shared by broader classes of disorders (e.g., anxiety disorders).

## Recent advances in improving the assessment of SAD

### Self-report measures

Even though numerous valid and reliable self-report measures are available for SAD, two new measures that addressed gaps in the available measures have emerged in recent years. The Socially Anxious Rumination Questionnaire (SARQ)^44^ is a 24-item measure assessing negative thoughts before (pre-event) and after (post-event) distressing social situations. The initial validation study showed promising results for its utility assessing rumination both before and after social interactions, which has long been identified as a key maintaining factor for social anxiety symptoms. The Ryerson Social Anxiety Scale (RSAS)^45^ is a 23-item measure developed to assess the distress and impairment associated with SAD, thus providing important information beyond most existing scales, which tend to focus on how severe and frequent the symptoms are without assessing broader impairment. A follow-up study has confirmed its validity and reliability in a clinical sample of patients with social anxiety^46^. These studies highlight the growing trend in clinical research and practice to move beyond simply assessing symptoms but to also assess maintaining factors that may be promising targets for treatment as well as to assess impairment and quality of life, which are considered patient-centered outcomes.

In addition, further validation efforts on existing scales have occurred in recent years. For example, a large-scale psychometric investigation provided further validation of the Multidimensional Social Anxiety Response Inventory – 21 (MSARI-21)^47^; the validity and reliability of the Self-Rated Beliefs in Social Anxiety (SBSA) scale were confirmed in a clinical sample^37^; an investigation in Brazil provided compelling data that translations of the widely used Liebowitz Social Anxiety Scale are valid and reliable tools for assessing social anxiety in global Spanish- and Portuguese-speaking individuals^48^; and data from large clinical and non-clinical samples confirmed that the computerized adaptive version and static short-form version of the widely utilized Social Interaction Anxiety Scale (SIAS) and Social Phobia Scale (SPS) are as valid and reliable as the original, lengthier versions^49^. Investigations such as these are critical to ensure that we have valid and reliable tools that assess all relevant aspects of social anxiety and do so in a manner that is culturally appropriate and brief for use in busy clinical and research settings.

### Leveraging technological advances to improve assessment of SAD

Clinical interviews and self-report measures will undoubtedly continue to play a critical role in diagnosing SAD and measuring change in severity of SAD symptoms over time. However, several researchers are leveraging recent technological advances to develop novel methods for assessing the disorder. One such method involves using data passively gathered from an individual’s smartphone (e.g., social interactions, geospatial movement, and activity level) to predict symptom trajectories. Jacobson and Bhattacharya^50^ recently found that such data could be utilized in personalized deep learning models to predict future symptoms of anxiety, as well as hour-to-hour changes in anxiety symptoms, among individuals with scores in the clinical range of well-validated self-report measures for SAD and generalized anxiety disorder (GAD). New methods for SAD assessment are also emerging from advances in machine learning, which refers to the use and development of computer systems that are able to learn and adapt without explicit instructions by using algorithms to analyze and draw inferences from patterns of data. A recent investigation by Demetriou and colleagues^51^ utilized sophisticated machine-learning algorithms to correctly differentiate three clinical conditions related with social difficulty: SAD, autism spectrum disorder, and prodromal psychosis. These studies provide excellent examples of the new frontiers that are being explored with respect to the assessment of SAD and other highly prevalent and impairing disorders.

## Recent advances in improving the treatment of SAD

### Digital therapy

As described above, CBT already has a solid base of evidence. Therefore, advances in the improvement of CBT for SAD in recent years have largely emphasized ways to increase access to treatment as well as understanding moderators and mediators of treatment outcome in order to inform treatment personalization and targeting of presumed mechanisms, respectively, both of which should improve outcomes.

One of the largest areas of contribution in the past few years has been in the area of employing digital therapy or virtual treatment for SAD. Digital therapy has the benefit of increasing access to care by reducing traditional barriers that can make coming into a clinic on a weekly basis difficult. However, findings from recent studies specifically evaluating digital therapy for SAD typically were limited by high attrition and weak comparison conditions. One study found that nine sessions of unguided internet-based therapy for SAD showed moderate effects on measures of social anxiety compared with waitlist control^52^. Although attrition from the study *assessments* was generally low, nearly half of the participants stopped completing sessions by session five out of nine, suggesting that most did not receive the full course of treatment. This is particularly notable given that the intervention did not introduce behavioral experiments or *in vivo* exposure until the last few sessions, so the intervention that most participants received was cognitive restructuring, thought to be efficacious but a less potent treatment ingredient than exposure for SAD. On the one hand, low treatment adherence/completion could be considered one of the many “failures” to retain patients through a prescribed course of CBT for SAD using self-guided, digital therapy. Indeed, attrition rates tend to be high for self-guided, app-based therapy^53^. On the other hand, patients showed significant and reliable improvement in SAD symptoms fairly rapidly, suggesting that perhaps patients self-discontinue treatment when they have achieved the benefits they aim to receive. Future studies with larger samples can elucidate dose–response relationships and the patterns that characterize treatment discontinuation and symptom improvement.

Similarly, Powell and colleagues^54^ evaluated a web-based self-help intervention for social anxiety and compared it with a waitlist control group. Even with an inactive control group, the improvements in social anxiety symptoms and cost-effectiveness (one presumed strength of self-directed interventions) were small. A web-based, self-guided, seven-session SAD intervention showed large effects on social anxiety symptoms (in an open trial) but only among those who completed treatment^55^. Attrition was also high in this study; only 38% of participants got to the fourth module, when behavioral experiments began. Similar to the study by Kahlke and colleagues^52^, this suggests that most people received cognitive restructuring but discontinued the self-guided treatment before any behavioral experiments or exposures began.

Collectively, these recent findings in web-based and digital therapies for SAD point to several issues that need to be addressed. Despite their proliferation and the enthusiasm that they bring to researchers and clinicians in terms of potential accessibility and cost-effectiveness, web-based and digital therapies must be delivered in ways in which patients adhere to them and see significant benefit. One promising approach may be in the area of digital approaches that utilize personalized engagement strategies, such as text messaging. Indeed, a brief text messaging intervention that encouraged patients to eliminate safety behaviors outperformed a comparison text messaging condition that encouraged individuals to focus on the present moment on social anxiety symptoms^56^. Another promising approach may be to directly target this lack of engagement and low treatment seeking in this population using web-based approaches. Indeed, a randomized trial of brief online interventions to increase treatment engagement in individuals with social anxiety found that a single-session online intervention that provided education and motivational strategies to individuals self-reporting social anxiety symptoms resulted in a significant uptake of treatment services^57^. Although this study did not have the benefit of comparison with a control condition that received no intervention (i.e., the conditions both included some form of intervention and both showed uptake increases) and thus it is difficult to draw conclusions about how these rates of uptake may differ from those of the general population of individuals who self-report social anxiety symptoms in the context of enrolling in an online study for individuals with social anxiety, this is nonetheless a promising finding and is particularly notable given its brevity and thus potential scalability. Future research should focus on strategies to increase engagement in digital therapy for SAD. This may include personalized feedback, live coaching via videoconference or text messaging, or adapting interventions so they are personalized and tailored to the individual’s needs in real time. Of note, some studies prior to the window of this review (i.e., before 2019) have explored some of these engagement strategies, particularly coach- or therapist-assisted digital platforms, and have demonstrated that this is a promising strategy for engagement in digital therapy and is deserving of future research.

### Novel approaches to delivering cognitive behavioral therapy for SAD

In addition to utilizing technology for the delivery of interventions for SAD, technology has recently been leveraged in novel ways. One study (a small pilot) evaluated the efficacy of ACT (a third-wave behavior therapy and efficacious treatment for SAD) delivered via videoconferencing for public speaking fear^58^. Virtual audiences were video recorded for homework exposures, a unique way to overcome the barrier that many CBT clinicians face in developing realistic, repeatable, individualized, and generalizable exposures for homework in this population with public speaking fear. Importantly, there were no statistically significant differences between those who received *in vivo* exposure versus those who received only the teleconference-based exposure, suggesting that this may be a viable alternative for patients with public speaking fears who do not have access to audiences or venues with which to practice public speaking exposures. However, these findings should be interpreted cautiously, given the small sample size. Replication with larger samples may be able to detect differences between two active conditions, which is difficult to do with small samples.

Other studies have recently used virtual reality to conduct exposure therapy for SAD^59,60^. Benbow and Anderson^59^ used a group therapy modality and showed significant long-term reductions in threat probability and cost estimates (i.e., two prominent cognitive mediators in CBT for SAD). Geraets and colleagues^60^ reported findings from a small, uncontrolled pilot study of virtual reality CBT for SAD and demonstrated improvements in SAD symptoms. These studies point to the exciting use of technology that should be explored further to develop ways to overcome some of the barriers to delivering *in vivo* exposure for patients with social anxiety, particularly those with performance-related anxiety that requires large groups of people to serve as an “audience” for exposure. However, randomized clinical trials with stringent control groups and larger sample sizes are needed to strengthen the rigor of this line of research.

### Addressing comorbidity

SAD is highly comorbid with other psychopathology, specifically MDD^61^ and substance use disorders (SUDs)^62^. As such, research that aims to better understand how treatment works for patients with these comorbidities, as well as how to improve upon treatments for patients with these comorbidities, can make a significant impact. A recent innovative study developed and evaluated a novel behavioral intervention that fully integrated treatment for SAD and AUD^63^. Patients were randomly assigned to a typical evidence-based intensive outpatient program for SUDs in a community-based SUD setting or to an intensive outpatient program that integrated CBT for SAD into the typical content and adapted the content to meet the specific needs of this population. The group therapy format common in SUD specialty clinics, which typically is seen as a barrier to successful engagement in addiction treatment for patients with SAD, was instead viewed as an opportunity to practice exposure in the context of alcohol recovery activities. Findings demonstrated superiority of the integrated treatment over the traditional treatment on social anxiety and some measures of alcohol use. These findings pave the way for integration of CBT for social anxiety into the settings where it is often prevalent but inadequately treated. Along similar lines, a recent study compared treatment for AUD only with an integrated treatment for SAD and AUD that included CBT and motivation interviewing^64^. Findings were somewhat similar: both conditions showed improvement in social anxiety and alcohol use, and the integrated treatment was superior to the control condition. However, no between-group effects were observed on alcohol use outcomes in this study. Taken together, these findings illustrate the importance of continuing to refine, improve, and augment these integrated interventions to simultaneously address this prevalent comorbidity.

In addition to this recent work, a research group has been exploring the role of depressive symptoms and MDD diagnoses on SAD treatment outcomes. One study found that, among those with a MDD diagnosis, change in social anxiety and depressive symptoms did not mediate change in each other but that change in social anxiety symptoms mediated change in depressive symptoms among those who had SAD and no MDD^65^. These findings suggest that change in social anxiety may lead to change in depressive symptoms among those with milder depressive symptoms, whereas this relationship was not observed at higher levels of depressive severity. Interestingly, and perhaps at odds with conventional wisdom, these researchers uncovered that depression was associated with better social anxiety outcomes among those with SAD who received treatment (broadly including individual CBT, internet-based CBT, group CBT, or pharmacology)^66^. The effect of greater depressive symptoms on better social anxiety outcomes was particularly large for those who had received individual CBT or internet-based CBT (but not for group CBT or pharmacology). These findings are particularly interesting as they call into question typical misconceptions that many community clinicians hold about CBT for anxiety disorders as being most appropriate, suitable, and effective for those without complex comorbidity. Indeed, patients with the common comorbidity of depression and SAD see significant benefit from a course of CBT for SAD.

Taken together, the last few years have generated only a few new articles specifically focused on treatment for SAD in the context of common comorbidities; but those few studies have yielded interesting findings with practical and direct clinical implications.

### Mediators and moderators of treatment outcomes for those with SAD

In addition to the studies described above that have touched on mediators of treatment outcome and change in presumed mechanisms of change^59,65^ and moderators/predictors of treatment outcome^66^, studies in recent years have attempted to understand for whom CBT for SAD works best (moderators) and the mechanisms by which change may occur during treatment (mediators). These fine-grained analyses provide clear prescriptive information for treatment selection based on individual difference characteristics and inform researchers about how to augment or modify treatments to directly address treatment targets that are the presumed mechanisms of change.

In a comparison of CBT with ACT for SAD, Sewart and colleagues^67^ examined changes in negative and positive affect in both conditions and also evaluated whether baseline negative and positive affect moderated treatment outcomes (i.e., differentially affected treatment outcomes between conditions). Negative affect decreased in both conditions, positive affect increased in both conditions, and neither negative nor positive affect at baseline moderated social anxiety outcomes. These findings indicate that ACT and CBT have common mechanisms despite their distinct theoretical approaches.

Another interesting study evaluating mechanisms of change asked an age-old question among CBT clinical researchers: “Does changing cognitions change behavior, or does changing behavior change cognitions?” Using a sample of patients with SAD who received CBT, the study found that avoidance behavior predicted cognitions at subsequent timepoints but not vice versa^68^. This is a fascinating finding showing the direct pathway from behavior to cognition but not the opposite, which is the more traditional way that the cognitive model is presented to patients. However, it is in line with a solid body of work showing that exposure therapy without any cognitive strategies works directly on changing threatening associations with feared stimuli (see Hofmann^69^ for a review).

Other interesting studies that aim to better understand how treatments work have observed that sudden gains in CBT and mindfulness-based interventions for social anxiety do not predict better outcomes by the end of treatment^70^, added to the existing literature supporting the importance of targeting social cost as a cognitive maintaining factor and finding that decreases in social cost estimates during exposure are associated with better social anxiety outcomes^71^, and identified that changes in shame mediated outcomes during exposure for SAD^72^. Additionally, a unique study auditing charts from real-world clinic patients found that typical treatment mediators observed in CBT for SAD in research settings also demonstrated significant mediation, including self-focused attention, negative social cognitions, and depressed mood^73^.

Finally, an interesting moderator study categorized participants as high or low in attentional biases at baseline^74^. These participants were randomly assigned to attention bias modification training (an alternative to CBT; see Hakamata et al.^75^ for a review) or placebo. Findings revealed no pre- to post-treatment effects on attention biases or anxiety in the condition that received the active intervention, and baseline attention biases did not moderate treatment. This study adds to the growing body of literature observing either null or small effects for attention bias modification for SAD^76^, which is unfortunate given the enthusiasm and promise it showed about a decade ago when it was developed and began to be evaluated as a low-intensity treatment option for anxiety disorders. Moreover, the fact that the intervention did not change the presumed mechanism of change is concerning.

## Future directions, recommendations, and conclusions

As illustrated above, SAD is an example of a fairly well-understood disorder with efficacious treatments. Yet, in a span of just three years, a tremendous amount of work has been done to improve our understanding and treatment of this prevalent and disabling disorder. Common themes and current directions in this work include (1) understanding the role of, assessing, and targeting cognitive and behavioral maintaining factors that contribute to the onset and maintenance of social anxiety; (2) understanding and treating common comorbidities with SAD, particularly AUD and MDD; and (3) evaluating strategies for increasing accessibility to evidence-based behavioral treatment for SAD through digital virtual and other technology-assisted modalities. Replication is needed for many of the etiological studies (with longitudinal designs when possible), and efforts are needed to improve engagement and retention in digital therapy programs. Nonetheless, much progress has been made in the understanding and treatment of SAD in the past few years. Future research should include randomized designs with larger samples that allow fully powered moderator and mediator analyses to truly identify factors that can optimize treatment outcomes for patients with SAD and common comorbidities.
